# Assessing Internet Quality Across Public Health Centers in Indonesia: Cross-Sectional Evaluation Study

**DOI:** 10.2196/65940

**Published:** 2025-09-15

**Authors:** Dewi Nur Aisyah, Agus Heri Setiawan, Alfiano Fawwaz Lokopessy, Chyntia Aryanti Mayadewi, M Thoriqul Aziz Endryantoro, Viktor Wibowo, Sarah Disviana, Indra Laksana, Mohammad Aviandito, Zisis Kozlakidis, Logan Manikam

**Affiliations:** 1 Department of Epidemiology and Public Health, Institute of Epidemiology and Health Care, University College London, 1-19 Torrington Place, London, WC1E 7HB, United Kingdom, 44 02076792000; 2 Digital Transformation Office, Ministry of Health Republic of Indonesia, Jakarta, Indonesia; 3 Monash University Indonesia, Banten, Indonesia; 4 Faculty of Medicine, Universitas Indonesia, Jakarta, Indonesia; 5 International Agency for Research on Cancer, World Health Organization, Lyon, France; 6 Aceso Global Health Consultants Pte Limited, Singapore, Singapore

**Keywords:** public health centres, public health, Puskesmas, primary care, primary health care, family physicians, primary care physicians, internet, web, internet connection, internet connectivity, internet access, infrastructure, community healthcare, technology, healthcare technology, digital health, medical informatics, evaluation studies

## Abstract

**Background:**

Primary health care centers (Puskesmas) serve as the cornerstone of Indonesia’s health care system, providing integrated services aimed at improving individual health through prevention, treatment, and health promotion. To fulfill these roles effectively, robust technological infrastructure, particularly reliable internet connectivity, is increasingly essential. Assessing the availability and quality of internet access in Puskesmas is therefore a critical step in understanding their readiness to implement digital health initiatives and fulfill their responsibilities in delivering accessible and effective healthcare services.

**Objective:**

This study provides a national baseline assessment of internet quality and its relevant IT infrastructure in more than 10,000 Puskesmas across Indonesia.

**Methods:**

A cross-sectional survey was conducted throughout all Puskesmas (10,382) in 34 provinces in Indonesia, using an online questionnaire. Categorization was done to analyze internet quality level results.

**Results:**

A total of 10,378 out of 10,382 public health centers (99.96%) participated in this study. Overall, 745 of 10,382 (7.18%) did not have internet access, 1487 (14.33%) had limited internet access, 5567 (53.64%) had sufficient internet access, and 2579 (24.85%) had sufficient and fast internet access. Moreover, 832 of 10,382 Puskesmas (8.02%) did not have 24-hour electricity, 44,196 (43.7%) had a central processing unit (CPU) with i3 specifications, 43,044 (42.56%) had 512 GB hard disk capacity, and 67,272 (66.5%) used antivirus.

**Conclusions:**

Although 79% (8201/10,382) of Puskesmas in Indonesia already had sufficient internet access, 21% (2180/10,382) still have limited and insufficient access. To ensure universal internet availability, it is essential to build collaborative support among internet providers and government to foster the availability and use of internet satellites, high-quality computers, and electrical power to support internet connectivity.

## Introduction

Primary health care (Puskesmas) is a core component of health systems [1]. Based on the Alma Ata Declaration in 1978, Puskesmas is defined as essential health care that meets the needs of communities, is easy to access, acceptable, and affordable [2]. The declaration combined 3 elements considered core to delivering sustainable and equitable health services: multisectoral public policy, empowered communities, and primary public health care [2,3].

In 2022, the Ministry of Health (MoH) of the Republic of Indonesia launched its health system transformation strategy. There are 6 pillars of the transformation strategy, and one of them is primary health care (PHC) transformation, which in its implementation focuses on strengthening promotive and preventive activities to create more healthy people, improve health screening, and increase primary service capacity. The number of Puskesmas has increased since 2017, from 9825 units to 10,374 Puskesmas in 2022. The ratio of Puskesmas in Indonesia to subdistricts in 2022 is 1.4. This illustrates that the ideal ratio of Puskesmas to subdistricts, namely a minimum of 1 Puskesmas in 1 subdistrict, has been fulfilled nationally. In terms of an average ratio, each Puskesmas serves 27,000‐30,000 residents in 2023. What needs to be considered is the distribution of the Puskesmas in all subdistricts, especially in remote, frontier, and outermost areas to provide public health services to households with a focus on maternal and child health services, elderly health, communicable disease, and noncommunicable disease prevention [4].

Puskesmas in Indonesia spread across all types of characteristic regions such as urban, rural, remote, and very remote in Indonesia within all 38 provinces and 514 districts or cities. In Indonesia, there are two main functions of Puskesmas: (1) provide an integrated individual health care that includes the activity and a series of basic individual medical services to improve the individual health, preventing and curing disease, and (2) provide essential public health services to the community, including health promotion, maternal and child health, nutrition, disease prevention and control, and environmental health. Puskesmas in Indonesia also has a responsibility to carry out technical guidance to clinics and other community health services as the Puskesmas networking partner institutions in the area. Other tasks of Puskesmas are to provide continuous and comprehensive care, to refer to specialists and hospital services, to coordinate health services, to guide the patient within the network of public health services, and to provide the best possible health services [5].

One of the elements that support health care services is IT. In the era of digital, Internet of Things, and artificial intelligence (AI), the digital transformation in health care also increases expectations of the quality of care [6]. Since the COVID-19 pandemic, digital information from the internet has gained importance in all areas of life. This trend was already evident before the COVID-19 pandemic. However, it accelerated this development and is also reflected in the health care sector [7]. With the widespread use of smartphones, downloadable or internet-based apps will play a major role in the diagnosis of diseases and monitoring and management of patients.

Technology and internet connection have played a significant role in bridging the gaps of health access and equity toward all individuals amid the geographic and socioeconomic challenges. In Indonesia’s decentralized health care system, Puskesmas are mandated to deliver not only curative services but also promotive, preventive, and administrative functions, particularly within the framework of the national health insurance program (Jaminan Kesehatan Nasional). Achieving these responsibilities requires functional coordination across various health system components, many of which increasingly depend on stable internet connectivity.

Internet access supports critical digital infrastructure for public health surveillance, insurance claims, medicine supply chain management, and national health program reporting. For example, real-time reporting through the Sistem Kewaspadaan Dini dan Respon and Sistem Informasi Malaria enables early detection and response to disease outbreaks—a crucial function in archipelagic and remote regions where delayed reporting can lead to localized epidemics [8]. Similarly, routine reporting on immunization services and noncommunicable disease screenings is now conducted through the Aplikasi Sehat IndonesiaKu, a digital platform mandated by the MoH for primary care reporting and national dashboard integration [9].

In addition, internet connectivity is required to operate pharmaceutical inventory management tools, such as e-Logistik, which help prevent medicine stockouts—one of the persistent challenges in rural and remote areas [10]. The national Cek Kesehatan Gratis campaign, a government-initiated free screening program for all Indonesian population, also requires digital reporting systems for real-time coverage tracking and participant data documentation [11]. Administrative functions, particularly processing health care claims via Indonesia Case-Based Groups under Badan Penyelenggara Jaminan Sosial Kesehatan, are highly dependent on reliable internet access to ensure service reimbursement and continuity [12]. Moreover, in the absence of specialist doctors in many rural settings, internet-based coordination with referral hospitals has become a crucial alternative for maintaining continuity of care. More recently, the implementation of the SATUSEHAT (ie, Satu Data Kesehatan) platform by the Ministry of Health requires Puskesmas to connect their electronic medical record (EMR) systems to a national health data exchange ecosystem, making stable internet access a prerequisite for full participation in Indonesia’s digital health integration efforts [13].

At the PHC level, internet connection has been a very critical infrastructure to support health data recording and reporting through a health information system, which is rooted from a routine data collection and serves as health indicators for government evaluations. These health data are critical to be disseminated to the health officials as evidence for program planning, and also to inform the patients related to immunization schedule, visit reminder, laboratory referrals, and other activities related to patients' follow-up [14].

Indonesia is the world’s fourth most populous country with more than 270 million people residing across more than 17,000 islands [15,16]. As an archipelagic country, this condition results in disadvantages in terms of information and communication technology (ICT) infrastructure [17]. In addition, almost all internet users in Indonesia connect through mobile devices. Although mobile broadband (3G, but now increasingly 4G/LTE) is the most widely used internet service in Indonesia, it is not at par with fixed broadband with respect to capacity, quality of service, high bandwidth performance, and cost-efficiency. Such limited access to high-quality internet prevents the population from unlocking its productive capabilities to fully reap the benefits of a digital economy and points to two key challenges: how to make fixed broadband internet access universal and increase internet quality. Increased optical fiber network coverage is a necessary but insufficient solution to meeting the key challenges. While investment by private service providers, along with government projects such as Palapa Ring, has steadily increased connectivity and service availability across Indonesian districts, internet subscriptions have not increased in parallel. Only 26% of homes with access to a fixed broadband provider subscribe to this service [18].

Several studies on IT infrastructure supporting health information systems in health care facilities have been conducted in Indonesia and other countries [19-21]. However, there is no detailed information available that can describe the accessibility and quality of the internet at Public Health Centers (Puskesmas) in Indonesia. This paper aims to provide, for the first time, a comprehensive overview of internet availability and connection reliability, as well as supporting IT infrastructure across Puskesmas throughout the country. While reliable internet connectivity is increasingly recognized as a foundational enabler for digital health transformation—including telemedicine, electronic health records, digital logistics, and surveillance systems—this study does not assess the direct impact of internet infrastructure on health care delivery or patient outcomes. Instead, it offers a baseline mapping of digital connectivity conditions to identify geographic disparities and inform future investments needed to enhance digital readiness in Indonesia’s primary care system.

## Methods

### Data Collection

A cross-sectional approach was conducted, using a survey conducted between November 15, 2022, and March 16, 2023. The population study covered 10,378 Puskesmas in 34 provinces in Indonesia. A questionnaire was used to collect data from participants. To ensure the validity and reliability of the questionnaire, a pilot survey was conducted with a total of 20 participants from Puskesmas across 5 major islands in Indonesia. This sample size aligns with established methodological guidance, which recommends 10‐30 participants for pilot testing to identify potential issues with instrument design and administration [22,23]. The pilot yielded a Cronbach α value of 0.76, indicating acceptable internal consistency [24] and supporting the reliability of the questionnaire items. These results provide a sound empirical and methodological basis for proceeding with the full-scale implementation of the instrument.

A standardized questionnaire survey was created using KoboToolbox (Kobo), an online application that allows users to create survey forms and collect data in real time. This tool was chosen because it can be accessed online or in offline mode (with automatic synchronization), even when the internet connectivity is poor. The tool also allows users to save drafts as they complete the surveys, giving them the flexibility to participate in surveys and carry out their daily tasks at Puskesmas. This is well suited to the uneven internet conditions in Indonesia.

The questionnaire was developed based on existing framework and literature, identifying aspects that may affect the internet quality. We adapted components from established internet quality assessment frameworks, primarily based on Quality of Service (QoS) and Quality of Experience (QoE) metrics, tailored to the infrastructure and operational context of Puskesmas in Indonesia [25-27]. The framework integrates both technical indicators (network performance) and infrastructure readiness (device and power availability). Network quality indicators (based on QoS) included type of internet connection (Wi-Fi, LAN, and routing), frequency and duration of internet disconnections, and bandwidth speed measured by Ookla speed test tool. In addition, we included device and environment indicators (infrastructure readiness): specifications and number of computers (CPU, RAM, and storage), availability of antivirus software, and electricity availability (uninterrupted power supply). To finalize the indicators, a series of discussions were carried out with experts from the infrastructure team at the Digital Transformation Office Ministry of Health Republic of Indonesia, IT specialists, public health professionals, and Public Health Governance team MoH. The final questionnaire (Multimedia Appendix 1) consisted of four sections, including (1) result of bandwidth speed test measured using Ookla speedtest tool in the morning and afternoon. Speedtest measures the speed between the device and the test server, using the Puskesmas device’s internet connection. (2) Type and quality of internet network such as Wi-Fi, LAN, routing, and so on, This section also provides qualitative information such as the condition of frequent internet disconnections and how the internet speed in Puskesmas. (3) Specification and quantity of computers or laptops in Puskesmas. This section collects information such as the capacity of CPU, memory, RAM, the total number of computers or laptops, antivirus installation, and the ownership of the computer or laptop. (4) Availability of electricity in Puskesmas. This section collects information on 24-hour electricity availability and any backup electricity used in Puskesmas.

Univariate analysis was conducted by open-source tools, using Python (Python Software Foundation) and R languages (R Core Team) version 3.10.0, to examine the internet quality in Puskesmas across Indonesia. Scoring was performed to determine the categories of internet quality. There were 11 principal component indicators used for scoring the quality of internet, including: (1) result of morning upload speed test; (2) result of afternoon upload speed test; (3) result of morning download speed test; (4) result of afternoon download speed test; (5) type of internet connection; (6) the quality of internet connection; (7) computer or laptop RAM specification used; (8) computer or laptop CPU specification used; (9) 24-hour electricity availability; (10) presence of backup or alternative electricity power supply; and (11) the number of laptops or computers with installed antivirus. Each indicator was assigned a maximum score and relative weight based on its potential contribution to the internet performance in PHC services. A weighted scoring table was used to rank the overall internet quality per facility. Indicators were given weights based on literature evidence and expert consensus. The detailed scoring calculation and weighting can be seen in Multimedia Appendix 1.

Final scores were divided into 4 categories, namely levels 1, 2, 3, and 4. Level 1 is for Puskesmas that have fast and reliable internet connection with 24-hour electricity and backup, as well as high specification of computer or laptop. Level 2 is for Puskesmas with normal and sufficient internet connection, standard specification of computer or laptop, and 24-hour electricity availability. Level 3 is for Puskesmas that have unreliable or insufficient internet connection, computer or laptop below standard, and lack of 24-hour electricity. The lowest level, level 4, is for Puskesmas with no internet connection, computer, or laptop below standard, minimum quantity, and lack of 24-hour electricity.

The major islands of Indonesia were classified into five categories: (1) Java Island, which includes provinces such as Banten, DKI Jakarta (Special Capital Region of Jakarta), West Java, Central Java, Yogyakarta Special Region, and East Java; (2) Bali and Nusa Tenggara, comprising Bali, East Nusa Tenggara, and West Nusa Tenggara provinces; (3) Sumatra, encompassing Aceh, North Sumatra, West Sumatra, Riau, Riau Kepulauan, Jambi, South Sumatra, Bengkulu, Bangka Belitung, and Lampung provinces; (4) Kalimantan, which includes West Kalimantan, Central Kalimantan, South Kalimantan, East Kalimantan, and North Kalimantan provinces; and (5) Maluku and Papua, consisting of Maluku, North Maluku, Papua, and West Papua provinces. After weighting each performance category of Puskesmas and understanding the demographic landscape of Indonesia, we used descriptive analysis, a statistical method used to summarize and describe the characteristics of a dataset to depict the condition of the internet in Indonesia.

The data cleaning procedure was carried out to ensure the accuracy and consistency of responses prior to analysis. First, all survey responses were screened for completeness; records with more than 20% missing values across key indicators were excluded. For records with limited missing values, we chose to analyze only complete cases (complete case analysis) for each variable to maintain transparency and avoid introducing assumptions about missing values. Given that the proportion of missing data was low and appeared to be randomly distributed, the risk of bias was minimal. While complete case analysis may reduce the sample size slightly, it ensures that the results are based solely on observed data, preserving the integrity of variable-level relationships and simplifying interpretation [28,29]. After calculating individual facility-level scores based on weighted indicators, we summarized the results at the provincial level by aggregating the total number of facilities in each internet quality category or selected indicators. The findings are presented descriptively to illustrate provincial-level distribution and variation. This approach allows for transparent comparison across provinces while preserving the granularity of facility-level scoring.

To minimize the risk of selection bias due to online survey assessment, we collaborated with the MoH Public Health Governance team working together with provincial and district health offices to ensure the survey completeness from Puskesmas. We allowed a 3-month period for survey submission for provincial and district health offices to have offline coordination with local Puskesmas, especially those located in remote and limited internet connectivity areas. We also conducted biweekly monitoring to ensure Puskesmas participation and completeness of the survey with provincial and district health offices.

### Ethical Considerations

We confirm that the data collected for this study did not require ethical approval, as no identifiable individual-level information was obtained or presented. Written informed consent was obtained from all participants prior to the commencement of the survey. The consent form explicitly stated that no personal or sensitive data would be collected and that all responses would be stored and analyzed in anonymized form. Participants were also provided with an information sheet outlining the study objectives and duration. No incentives or compensation were provided to participants.

## Results

### Internet Quality Across Puskesmas

A total of 99.96% Puskesmas (10,378/10,382 Puskesmas in Indonesia), spread across 514 districts or cities from 34 provinces, participated in the survey. Among the 34 provinces, all Puskesmas in 30 provinces (88%) successfully participated in this survey. Based on the location of Puskesmas, 29.2% (3034/10,378) were located in urban areas, 45.3% (4700/10,378) in rural areas, 13.9% (1446/10,378) in remote areas, and 11.5% (1198/10,378) in very remote areas. The detailed participation rate across Puskesmas in 34 provinces can be accessed from Multimedia Appendix 2

Based on the study results, 7.18% Puskesmas in Indonesia (745/10,378) had no internet access (level 4), 14.33% Puskesmas (1,487/10,378) had insufficient internet access (level 3), 53.64% Puskesmas (5,567/10,378) had sufficient internet access (level 2), and 24.85% Puskesmas (2,579/10,378) had sufficient and fast internet connection (level 1). Given this condition, 78% (8,097/10,378) Puskesmas in Indonesia, categorized under levels 1 and 2, exhibited sufficient internet access, whereas 22% (2,235/10,378) of Puskesmas in levels 3 and 4 experienced inadequate internet connectivity.

**Figure 1.**
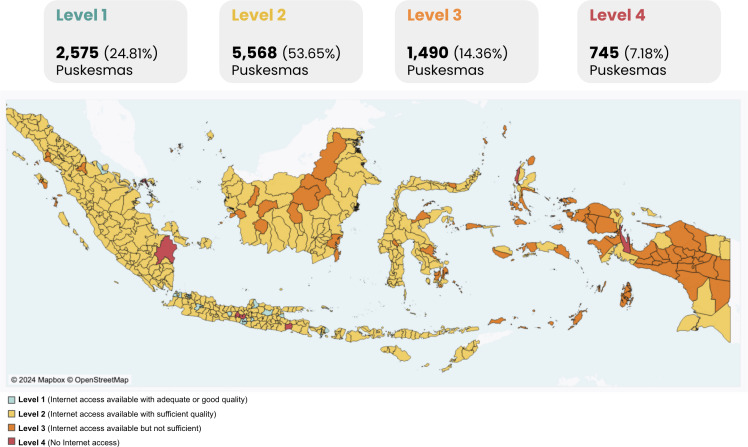
Distribution of Puskesmas internet quality across provinces in Indonesia.

After obtaining data on each Puskesmas, we calculated the level of internet quality at the provincial level by averaging the scores of all Puskesmas in each province (Figure 1). Provinces with the highest proportion of Puskesmas having level 1 internet access were DI Yogyakarta (86/121, 71%), followed by DKI Jakarta (206/315, 65.4%), and Bali (51/120, 42.5%). In contrast, 48.9% (222/454) Puskesmas in Papua, 48.9% (81/227) Puskesmas in Maluku, and 25.7% (42/163) Puskesmas in West Papua had the highest number of Puskesmas with no internet access in the province. Out of 745 Puskesmas without internet, the provinces with the highest number of Puskesmas with no internet access were Papua (222/745, 29.79%), Maluku (81/745, 10.87%), North Sumatra (45/745, 6.04%), West Papua (42/745, 5.63%), and North Maluku (35/745, 4.69%). Further details are available in Multimedia Appendix 3.

**Figure 2.**
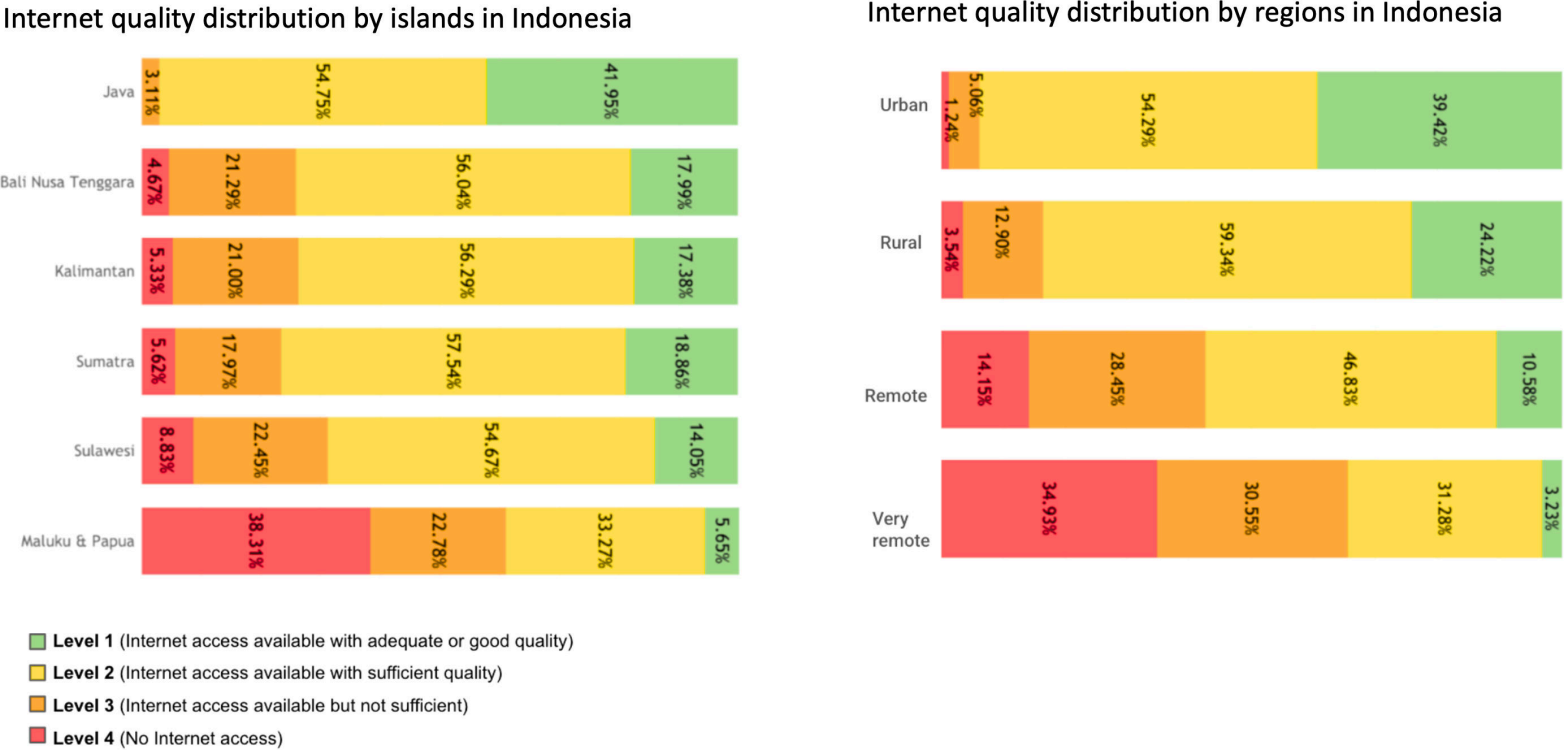
Distribution of Puskesmas internet quality based on islands and locations in Indonesia.

The disparities in the distribution of Puskesmas with different levels of internet quality across the islands of Indonesia were identified. Java Island led with a large number of Puskesmas having level 1 internet access (1525/3635, 41.95%), marking a stark contrast to other islands, such as Sumatra Island, Sulawesi, Kalimantan, Bali, and Nusa Tenggara, where the proportion of Puskesmas with fast internet access did not exceed more than 20%. Nationally, the level 2 internet category is the most common, with 53.64% (5,567/10,378) Puskesmas having sufficient internet access, and Sumatra Island having the highest proportion within this category (1,556/2,704, 57.54%). In sharp contrast, the Maluku and Papua Islands have the highest distribution of level 4 internet quality with 38.31% (380/992) Puskesmas having no internet access, which is significantly higher than other islands (Figure 2).

The distribution of Puskesmas with varying levels of internet quality is also significant across different locations in Indonesia. Puskesmas located in urban areas were more likely to have better internet quality, with 39.42% (1177/2986) Puskesmas having sufficient and fast internet access. Puskesmas located in rural areas were more likely to have sufficient quality internet with 59.34% (3004/5062) Puskesmas in level 2. Puskesmas in very remote areas were disproportionately affected by lower internet quality, with the highest occurrences of level 4 and level 3 internet quality (335/959, 34.93% and 293/959, 30.55%, respectively) (Figure 2).

Based on computer connection, majority type connections in Indonesian Puskesmas were Wi-Fi (5179/10,378, 49.9%), followed by Wi-Fi and LAN (2288/10,378, 22.05%), and tethering from mobile data (772/10,378, 7.44%). Referring to participants’ perspective, 38.83% (4030/10,378) Puskesmas had robust internet access, 31.83% (3303/10,378) had slow internet connection, 22.16% (2300/10,378) had very slow internet connection, and lastly 7.18% (745/10,378) had no access to internet at all (Multimedia Appendix 4). Type of internet connection and internet connection quality in public health centers have a weight of 8% each of the total assessment.

### Factors Influencing Internet Quality Across Public Health Centers

#### Computer Specification

A separate analysis was conducted to determine other factors that affect internet quality, including RAM specifications, CPU, hard disk capacity, and antivirus updates on the hardware used. Based on the study, the most common RAM specification used in Puskesmas was 4 GB RAM (61,390/101,140, 60.7%), followed by 2 GB RAM (17,536/101,140, 17.34%), and 8 GB RAM (17,038/101,140, 16.85%).

**Figure 3.**
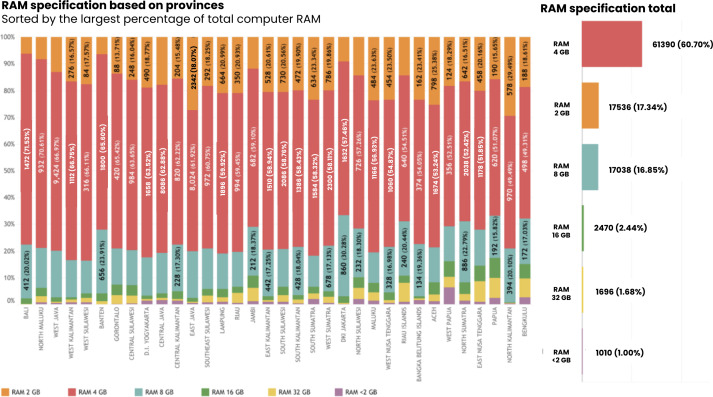
RAM specification based on provinces and total values.

Based on regional distribution, the provinces with the largest proportion of 4 GB RAM were Bali (1472/2058, 71.53%), followed by North Maluku (932/1320, 70.61%) and West Java (9942/14,072, 66.97%). In addition, East Nusa Tenggara had the highest proportion of Puskesmas with 23 GB RAM computer specification (174/2272, 7.66%), followed by Papua (90/1214, 7.41%), Riau Islands (84/1174, 7.16%), and Bengkulu (70/1010, 6.93%). On the other hand, West Papua Province was the province with the highest proportion of hardware with a low specification less than 2 GB RAM (44/678, 6.49%), followed by Bengkulu (28/1010, 2.77%), Papua (30/1214, 2.47%), and North Sulawesi (26/1268, 2.05%) (Figure 3).

**Figure 4.**
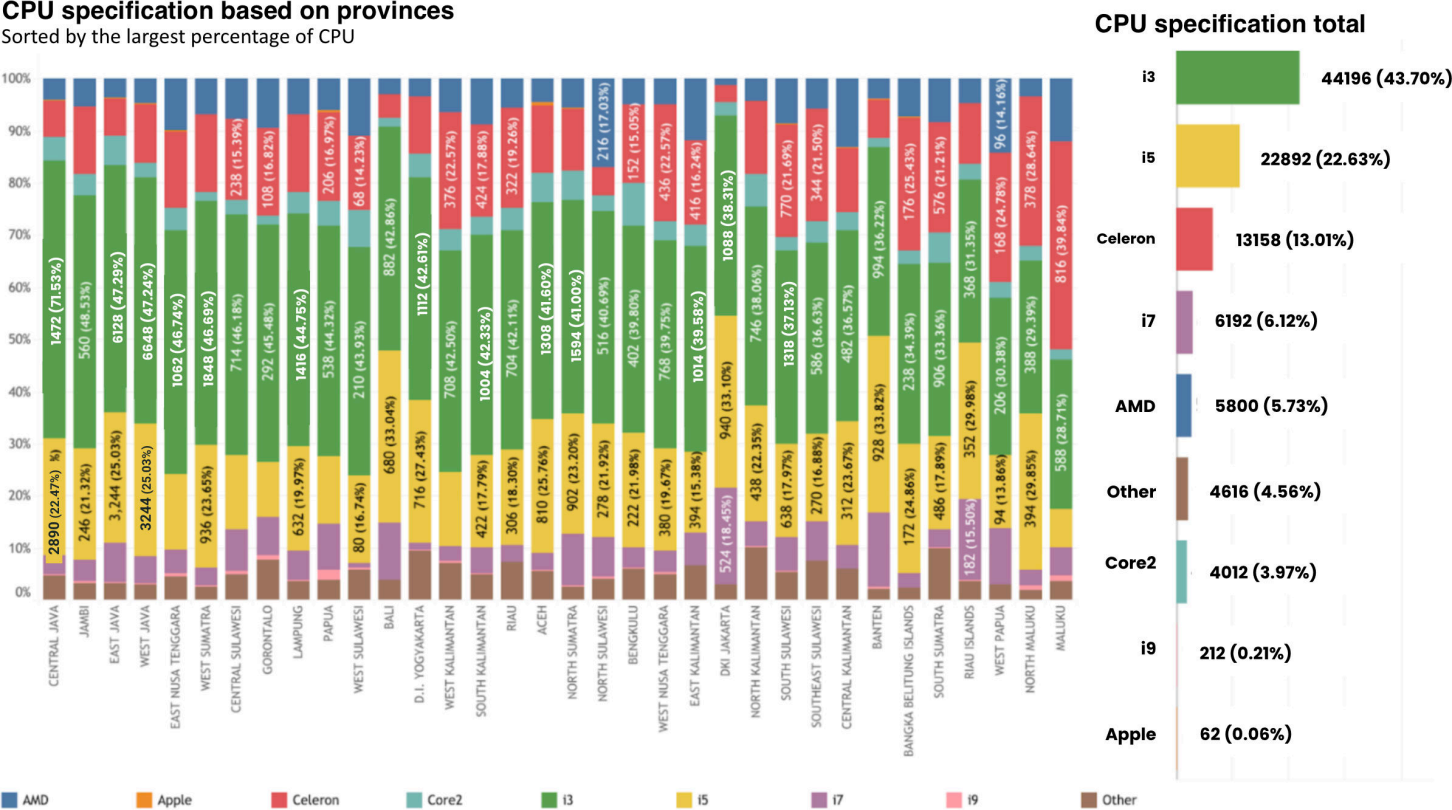
CPU specification based on provinces and at the national level.

By region, the provinces with the highest proportion of hardware with CPU type i3 were Central Java (6860/12,860, 53.34%), followed by Jambi (560/1154, 48.53%), and East Java (6128/12,958, 47.29%). In addition, provinces with the highest CPU specification of i7 type were DKI Jakarta (5242/2840, 18.45%), Riau Islands (182/1174, 15.5%), and Banten (390/2744, 14.21%). In contrast, the provinces that had below-average CPU type distribution with Celeron type were Maluku (816/2048, 39.84%), followed by North Maluku (378/1320, 28.64%) and Bangka Belitung (176/692, 25.43%) (Figure 4). Based on CPU specifications, nationally, hardware with i3 specifications dominated at 43.7% (44,196/101,140), followed by i5 at 22.63% (22,892/101,140), and Celeron at 13.01% (13,158/101,140) (Figure 4).

**Figure 5.**
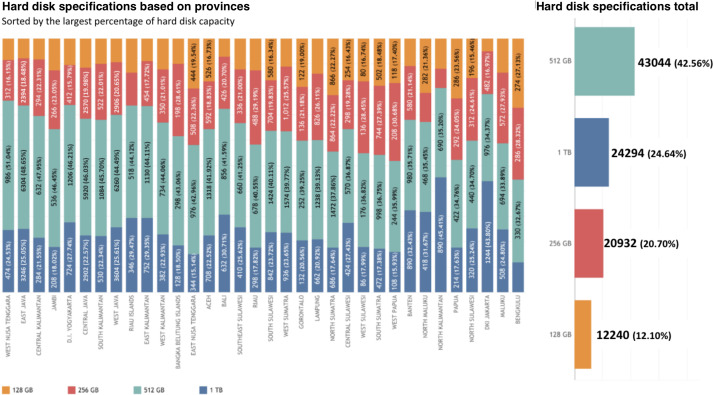
Hard disk specification based on provinces and at the national level.

At the province level, the provinces with highest distribution of 512 GB hard disk capacity were West Nusa Tenggara (986/1932, 51.04%), followed by East Java (6304/12,958, 48.65%), Central Kalimantan (632/1318, 47.95%), and Jambi (536/1154, 46.45%). In addition, provinces with the highest proportion of highest hard disk capacity of 1 TB were North Kalimantan (890/1960, 45.41%), followed by DKI Jakarta (1244/2840, 43.8%), Banten (890/2744, 32.43%), and North Maluku (418/1320, 31.67%). In contrast, the province with the highest distribution of lowest hard disk specification of 128 GB was Bengkulu (274/1010, 27.13%), followed by Papua (286/1214, 23.56%), North Sumatra (866/3888, 22.27%), and North Maluku (282/1320, 21.36%). The details can be seen in Figure 5; it shows that nationally, the most hard disk capacity used in Puskesmas was 512 GB (43,044/101,140, 42.56%), followed by 1 TB (24,924/101,140, 24.64%), 256 GB (20,932/101,140, 20.7%), and 128 GB (12,240/101,140, 12.1%).

**Figure 6.**
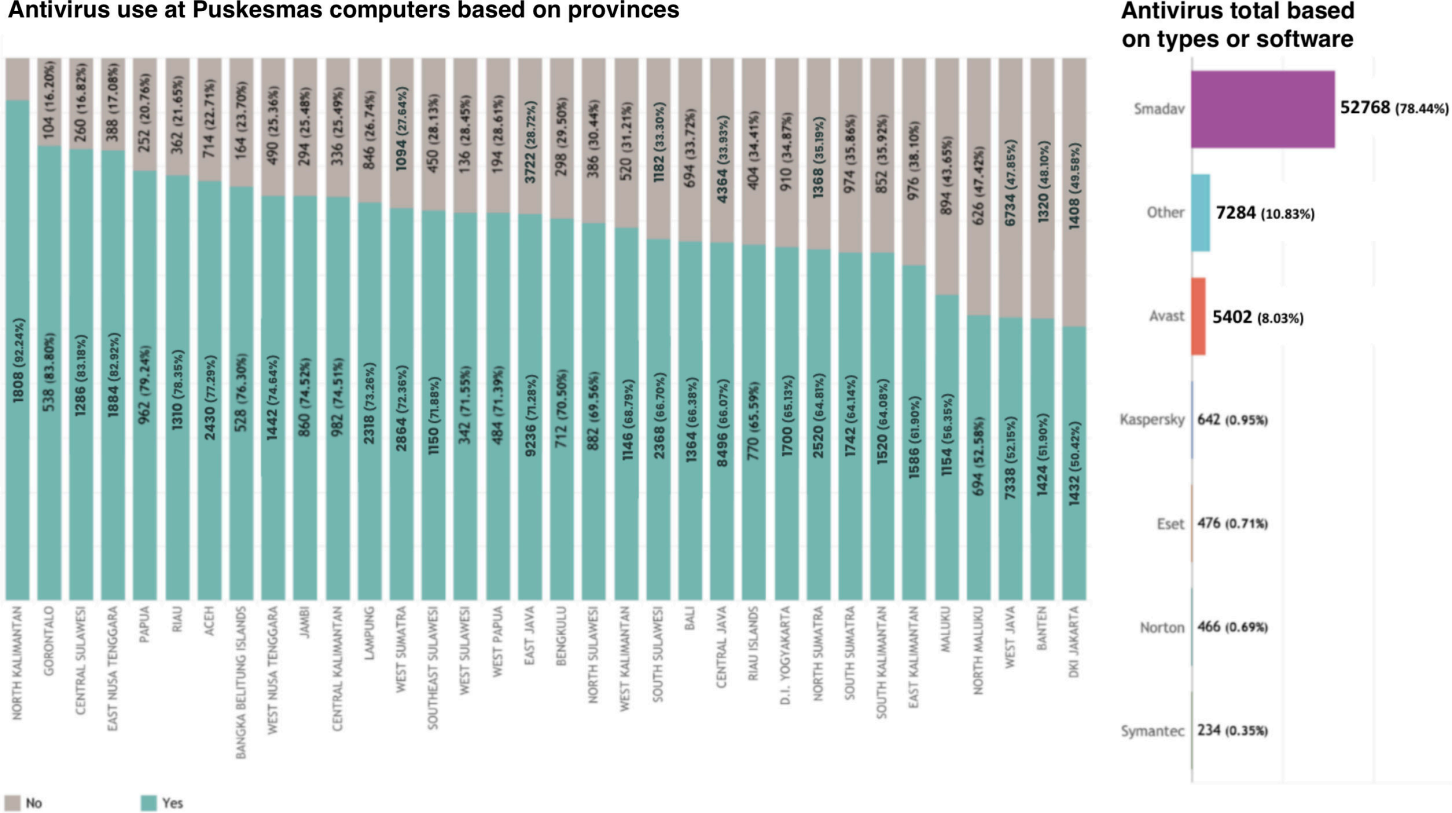
Antivirus use at Puskesmas computers based on provinces and on types or software.

The province with the highest proportion of computers with installed antivirus was North Kalimantan (1808/1960, 92.24%), followed by Gorontalo (538/642, 83.8%), Central Sulawesi (1286/1546, 83.18%), and East Nusa Tenggara (1884/2272, 82.92%). In contrast, DKI Jakarta, with 49.58% (1408/2840) of their computers, did not install antivirus, followed by Banten at 48.1% (1320/2744), West Java at 47.85% (6734/14,072), and North Maluku at 47.42% (626/1320) (Figure 6). Based on the study, 33.5% (33.868/101,140) of computers in Puskesmas did not install antivirus. Among the computers with installed antivirus, the most antivirus used was Smadav (52,768/67,272, 78.44%), followed by Avast (5402/67,272, 8.03%), and others (7284/67,272, 10.83%) (Figure 6).

#### Electricity Access

**Figure 7.**
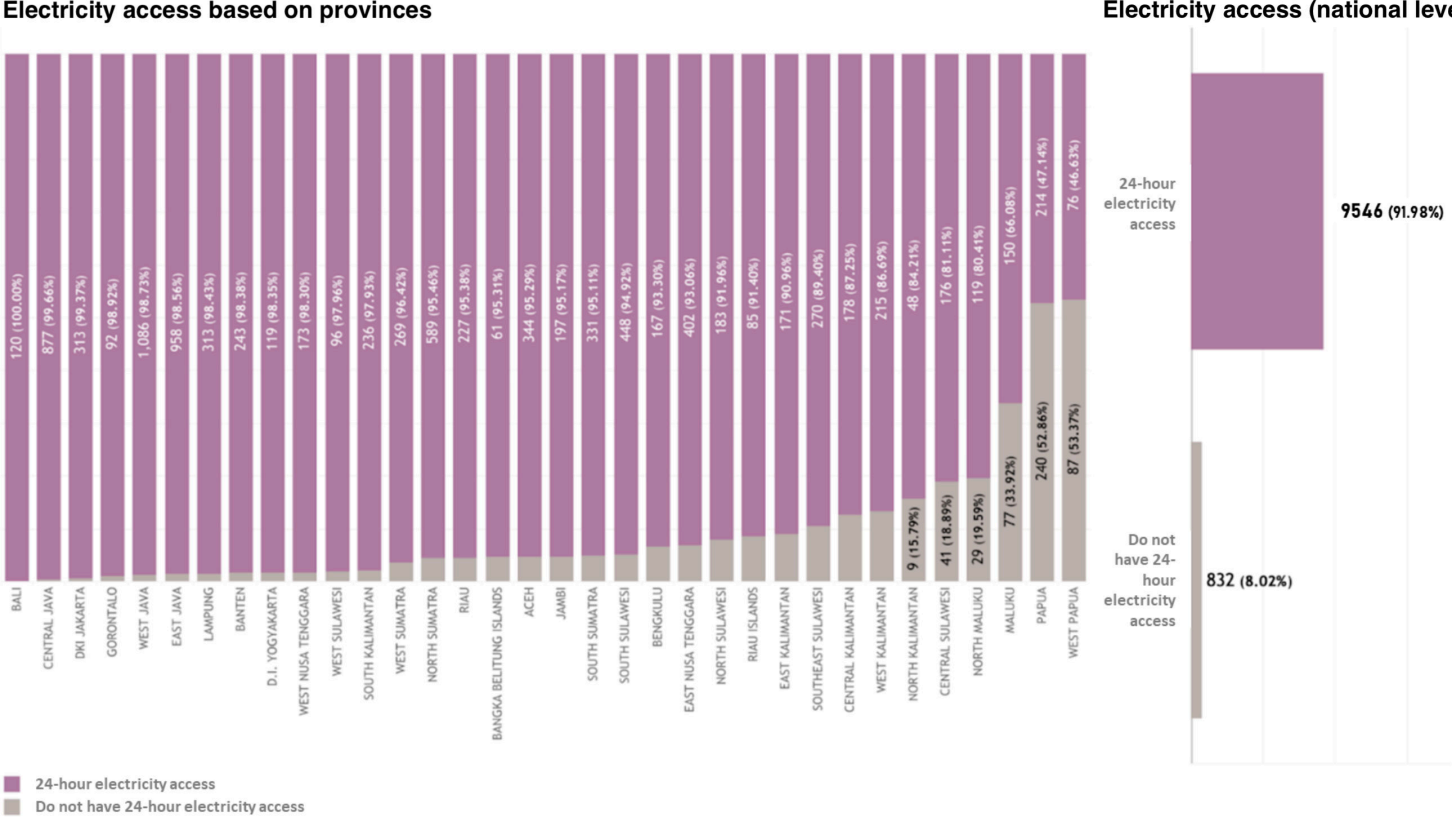
Electricity access based on (A) provinces and (B) at the national level.

In terms of province distribution, 100% (120/120) Puskesmas in Bali have access to 24-hour electricity, followed by Central Java (99.66%, 877/880), DKI Jakarta (313/315, 99.37%), and Gorontalo (92/93, 98.92%). In contrast, provinces with the highest number of Puskesmas that do not have access to 24-hour electricity were West Papua (87/143, 53.37%), followed by Papua (240/454, 52.86%), Maluku (77/227, 33.92%), and North Maluku (29/148, 19.59%) ( Figure 7A). At the national level, 91.98% (9546/10,378) Puskesmas in Indonesia have electricity access for 24 hours, whereas 8.02% (832/10,378) Puskesmas do not have access to 24-hour electricity (Figure 7B).

**Figure 8.**
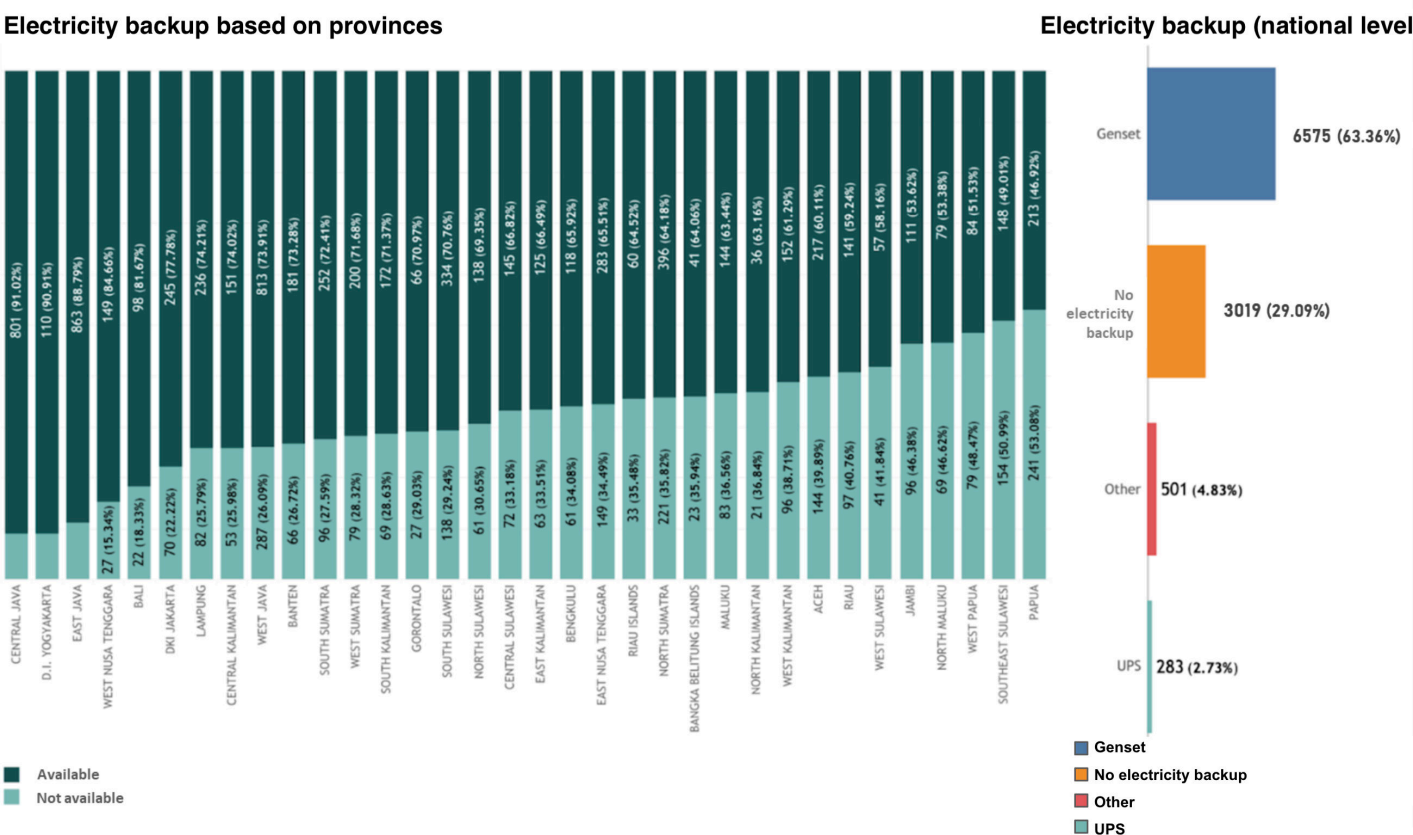
Electricity backup based on (**A**) provinces and (**B**) at the national level.

The provision of electricity backup is also an important point in the operational process of public health centers. Based on the study, 29.09% (3019/10,378) of Puskesmas did not have electricity backup (Figure 8). Among the Puskesmas who had electricity backup, the most common type of electricity backup owned by Puskesmas was a generator (6575/10,378, 63.36%), followed by other backups (501/10,378, 4.83%), and UPS (uninterrupted power supply; 283/10,378, 2.73%). At the provincial level, the provinces with the highest number of Puskesmas having electricity backup were Central Java (801/880, 91.02%), followed by DI Yogyakarta (110/121, 90.91%), East Java (863/972, 88.79%), and West Nusa Tenggara (149/176, 84.66%). In contrast, 53.08% (241/454) Puskesmas in Papua did not have any electricity backup, followed by South East Sulawesi (154/302, 50.99%), West Papua (79/163, 48.47%), and North Maluku (69/168, 46.62%).

## Discussion

The internet’s significant role in aiding public health services is undeniable. This paper marks the first comprehensive depiction of the accessibility and quality of internet connections and computer systems across Indonesia’s Puskesmas, providing crucial insights into the information technology infrastructure. The internet availability in the health facility is highly crucial to foster the delivery of healthcare among communities [28,29]. By presenting baseline data on internet quality across Puskesmas, this study contributes foundational insights to support future efforts aimed at strengthening health information systems and expanding the digital infrastructure needed for more equitable and efficient PHC delivery in Indonesia.

Indonesia presents a particularly interesting case when it comes to internet connectivity and accessibility to health care services, especially in the context of health care digitization [30]. In numerous instances, technological advancements have the potential to significantly reduce health care inequalities. Furthermore, digital health is recognized as a valuable technological approach that can effectively bridge the significant gap between urban and rural populations [31] and between high-income and low- and middle-income countries (LMIC) [32].

One way of improving health care service delivery is through adoption of broadband Internet access [33]. In the health system, service delivery refers to where patients receive the treatment as well as supplies entitled. Broadband internet access helps to reshape many health-related processes and supports effective treatment of patients [33]. It helps medics to provide patients with faster diagnosis to their respective health conditions, the development of treatment plans, making patient records as well as test results accessible by patients from the examination room, facilitates faster consultation electronically on treatment plans, helps patients and their attendants access practice guidelines faster [15,34,35], and led to the adoption of telehealth technologies which has contributed to easy access to health services [36]. The availability of Internet access, coupled with the accelerated adoption of eHealth and mHealth platforms and the rapid evolution of digital technology access, has generated a significant opportunity to enhance the reach, quality, and efficiency of PHC service delivery, thereby facilitating the achievement of universal health coverage [37].

According to this study, it was found that 78.4% (8,143/10,378) of Puskesmas have good and sufficient internet quality, 14.4% (1,490/10,378) Puskesmas have insufficient internet connection, and 7.2% (745/10,378) of the Puskesmas do not have internet access at all. The study depicts that the eastern part of Indonesia had the least internet connection in Puskesmas compared to other parts of Indonesia, which includes Maluku and Papua regions. This is aligned with previous findings related to the limited internet access in the eastern part of Indonesia and low ICT development index in 2020‐2021 throughout the areas [38-41]. The ICT Development Index, published by the United Nations International Telecommunication Union, serves as a crucial benchmarking tool for measuring the information society by utilizing internationally agreed ICT indicators, assisting governments, operators, development agencies, researchers, and others in assessing the digital divide and comparing ICT performance globally [42]. Poor internet connectivity is one of the barriers to implementing telehealth [43]. Studies reveal varying rates of internet connection across different countries affect the adoption and implementation of telehealth and eHealth [44,45]; and digital health initiatives, especially in rural areas [46]. The underlying causes of limited internet access in the eastern part of Indonesia may be due to several aspects, including challenging geography, archipelagic region, size of territory to cover, limited infrastructure, which should be taken into account as barriers that limit the accesses [47-49]. High service costs to provide IT infrastructure (including tower, fiber optic, etc) have become the challenge to evenly provide internet access across the country, especially in remote areas [50]. While at the same time, the user also finds it a burden to subscribe to the certain internet monthly fee [51-53]. Some studies also showed that sociobehavioral values and perceptions toward digitalization also influence enhancing or limiting the internet availability, including unwillingness of the local people to allow the internet infrastructure development, lower education attainment, and low adult literacy [52,54].

For completing administrative tasks in Puskesmas, such as browsing, data input, upload and download files, or to support any other health service tasks, according to information and technology literatures, it requires a minimum specification of 4 GB RAM and CPU i3 or above for computer or laptop to perform basic data management (input/edit/delete data or simple online browsing) [55,56]. These findings show that the majority (60.70%, 61,390/101,140) of the Puskesmas in Indonesia already had 4 GB RAM or above in their computers, while 18.34% (18,546/101,140) of them were lower (2 GB and 1 GB). As for the CPU specification, 83% (83,970/101,140) of the Puskesmas had the sufficient CPU specification (i3 type or above), while 17% (17,170/101,140) of them had insufficient, which were Celeron and Core2. It is understood higher RAM capacity and newest CPU specifications are linked with better computer performance, which is highly important to be provided in Puskesmas to ensure smooth operation of critical health care software, efficient management of electronic health records, and reliable services, ultimately supporting effective patient care delivery in the health care facilities [57,58].

Although CPU, RAM, and memory do not directly control bandwidth or download speeds, they significantly influence the user’s ability to use the internet efficiently for digital health service. The CPU determines how quickly a computer can process incoming data from the internet, execute scripts, and render web pages. A slower CPU may lead to delays in page loading, application responsiveness, and user interaction, particularly with web-based health information systems. Goel et al [59] demonstrated that lowering CPU performance (eg, through power-saving modes on Android devices) results in slower page loading and degraded user experience in accessing online resources. Similarly, another study notes that a slow processor can act as a bottleneck when decoding or rendering content received over the internet, making the browsing experience sluggish [60]. RAM enables systems to temporarily store and quickly access active data. Systems with insufficient RAM often struggle when running multiple applications or browser tabs, leading to frequent slowdowns or crashes. This affects the usability of internet-dependent applications, especially in environments such as health centers where simultaneous tasks are common. While RAM does not increase raw internet speed, having adequate memory ensures smoother browser and application performance [61]. Another report supports this by showing that memory capacity and technology trends directly influence web system performance and throughput [62].

Similarly, with RAM capacity and processors, hard disk was also found to affect the computer’s capacity to process [58]. This study reveals that the majority of Puskesmas have a sufficient hard disk of 256 GB or above (88%, 20,932/100,510), and 12% (12,240/100,510) of them have an insufficient one (128 GB). Literature findings show that 128 GB internal storage is able to undergo basic operations; however, it will demand higher capacity for extensive computer use [63]. The hard disk is crucial for computer performance as it stores and retrieves data, including the operating system and applications, facilitating efficient multitasking and overall system responsiveness [57,58].

Across various low- and middle-income countries, the results consistently highlight significant disparities in hardware infrastructure at the PHC level, which directly impact the implementation of digital health systems [64]. In Nigeria, many PHC centers still rely on desktop computers with only 512 MB to 2 GB of RAM and outdated Pentium IV processors, limiting their ability to run modern eHealth applications [65]. Similarly, in Uganda, facilities were found using laptops with 1 GB RAM and low-speed CPUs, which caused frequent delays and hindered real-time data entry and synchronization [66]. In Kenya, a study revealed that more than half of the PHC sites were equipped with low-specification devices using Intel Atom processors and 2 GB RAM, often unable to handle multiple web-based platforms simultaneously [67]. In parts of Latin America, such as Peru and Bolivia, rural clinics were reported to operate with refurbished computers having less than 2 GB RAM, rendering them incompatible with many EMR systems [68]. These findings underscore the urgent need for targeted investment in basic digital infrastructure to ensure the successful adoption of health information systems in resource-constrained settings [69].

As for the antivirus, this finding shows that the coverage of antivirus installation in Puskesmas’ computers is still incomplete, especially in DKI Jakarta, Banten, and West Java provinces, which leave almost half of its Puskesmas unprotected with the antivirus. In health care facilities, malware protection is critical for computer performance as it safeguards sensitive patient data, prevents potential cyberattacks, and ensures the uninterrupted operation of vital medical software, maintaining the integrity and security of patient care services [70,71]. Several studies have highlighted the limited implementation of antivirus software and weak cybersecurity practices in PHC facilities across LMICs. Hasegawa et al [72] conducted a scoping review and found that antivirus installation in PHC settings was often inconsistent or absent due to limited funding, lack of technical support, and absence of standardized cybersecurity policies. Similarly, the World Health Organization (2021) reported that many LMIC PHCs do not regularly update or install antivirus software, making them vulnerable to malware and data breaches [73]. A scoping review study by He et al [74] found that one of the major challenges of healthcare cybersecurity is the lack of security awareness. Collectively, these findings underscore the urgent need for national digital health policies that mandate and support sustainable cybersecurity infrastructure, including antivirus software, within PHC systems across LMICs.

This study found that 8.02% (832/10,378) of Puskesmas in Indonesia have no access to electricity for 24 hours. Most provinces that did not have access to 24-hour electricity were located in the eastern part of Indonesia, such as Maluku and Papua island. Modern energy enables health service delivery. Access to electricity is, however, unreliable in many health facilities in developing countries [75]. Reliable basic infrastructure, particularly electricity, is a critical enabling factor in improving health systems [76]. Previous literature showed that the availability of electricity is an important determinant of receiving health information and use of health services [77,78]. Based on a literature study conducted in developing countries [65,], there are many barriers that can occur during the electricity provision across the nation, this includes the lack of IT infrastructure, especially electricity supply. Indonesia started the rural electrification program in the late 1950s, but how to provide electricity in a sustainable way both organizationally and institutionally still becomes a big challenge [79]. According to the World Health Organization (2023), nearly 1 billion people globally are served by health care facilities that either lack electricity altogether or rely on highly unreliable sources, with PHC centers being especially affected [78]. A systematic review by Ibrahim et al [80] found that fewer than 30% of PHC facilities in LMICs had consistent electricity access, which severely undermines the ability to store vaccines, operate diagnostic equipment, and provide emergency care [80]. In a multicountry study of surgical hospitals across 21 LMICs, continuous electricity availability was reported in less than two-thirds of facilities, with countries such as Sierra Leone and Malawi experiencing 0% reliable power supply [81]. These power gaps directly limit the deployment of digital health technologies, vaccine cold chains, and essential services. According to the Electricity Power Supply Business Plan 2019‐2028, Indonesia’s average electricity growth is at 6.42%. Indonesia successfully surpassed its electrification ratio target at the end of the year 2018 [81]. However, there are still 2510 villages across Indonesia that are not yet electrified [82]. Increasing the electrification ratio always becomes a challenging task because Indonesia has many islands, and many communities are isolated from one another [79]. This geographical situation also worsens with poor infrastructure access, making the area hard to reach [83]. There are several fundamental requirements that need to be implemented in order to cope with the rural electrification challenges [84-86]. The lessons learned from other countries that have successfully achieved universal electricity access were summarized in several studies [87]. Committed governance and supportive regulations and policies are one of the most often stated in the literature [87]. Moreover, a collaboration of appropriate policies and government will increase the private sector’s participation in the rural electrification program [88].

Based on this study and consultation with experts, it is recommended that Puskesmas facilities in Indonesia require a minimum internet bandwidth of 10 to 20 Mbps. This range is deemed as a minimum essential requirement to ensure reliable access to digital resources and the efficient input of patient EMR data. In addition, PHC services should be equipped with at least Intel Core i3 processors, 8 GB RAM, and 256 GB solid-state drive storage to enable smooth operation of digital applications and web-based platforms [69]. Optimal performance, especially in urban or high-volume facilities, may require upgrading to Intel Core i5 processors and 16 GB RAM, which align with the performance needs of multitasking systems and real-time data entry [67,68]. This study provides a structured and evidence-based framework for assessing internet quality in PHC facilities, which is especially relevant for benchmarking across comparable middle-income countries. Many countries with similar socioeconomic and infrastructural conditions face shared challenges in ensuring reliable digital connectivity in remote or underserved health centers [89,90]. By developing and validating a weighted, multi-indicator scoring system, grounded in technical, infrastructural, and operational factors, this study offers a replicable model that can inform digital health readiness assessments beyond Indonesia. Such benchmarking tools are essential as countries pursue universal health coverage and integrate eHealth systems in alignment with the World Health Organization’s Global Strategy on Digital Health 2020‐2025. Additionally, initiatives like the International Telecommunication Union and the World Bank’s Digital Economy for Africa and Asia programs emphasize the need for context-sensitive indicators to guide investments in health ICT infrastructure [91,92]. Therefore, this study not only addresses a national implementation gap but also contributes to the global evidence base supporting digital health infrastructure development in resource-constrained settings.

Successful implementation, rollout, and use of the digital system require PHC facilities to be equipped with appropriate digital and data infrastructure. In Tanzania, as part of the National PHC Rolling Digital Transformation Roadmap, service delivery points for in-facility continuity of care are using a LAN that is installed within facilities [93]. While interfacility referrals and integration with public domain systems. While in China, in order to guide the development of internet health care, the Chinese government has issued a series of regulations and guidelines. The State Council of the People’s Republic of China has issued the Guidance on Actively Promoting the Action of “Internet Plus.” It proposed to develop internet-based health care services, encourage internet enterprises to cooperate with medical institutions for establishing medical network information platforms, reinforce the integration of regional health care resources, and actively apply the mobile internet to explore and provide health services [94]. Depending on the different initiators, there are 3 main modes of internet health care service provision in mainland China: hospital-led, government-led, and enterprise-led internet healthcare platforms [95].

This research exhibits several strengths that contribute to its significance in the field. The study at hand is a groundbreaking research effort that has comprehensively assessed a wide range of internet components in PHC facilities, including device availability, access, and internet quality in nearly all public health centers across Indonesia. The findings of this study provide valuable insights into various supporting factors that can facilitate the implementation of IT transformation. The study highlights the significance of the availability, quality, barriers of computer systems, hardware, and software devices, which can serve as instrumental inputs for the government in their decision-making processes regarding the implementation of such transformations.

However, certain limitations must be acknowledged: (1) Bandwidth checks to assess the quality of internet access were only carried out in the morning and evening, which depends on local conditions and the respondent’s honesty; (2) the completeness of filling out the questionnaire was influenced by the capacity of the assigned respondents; (3) validation of CPU, RAM, and antivirus could not be done directly; (4) potential selection bias may occur as we performed the assessment using an online survey, although we have minimized this bias by having coordination with the designated Public Health Governance team MoH and district or Provincial Health Offices allowing them to complete the survey within a 2-month period, having offline coordination to ensure we obtained survey feedback from Puskesmas. Nevertheless, this study provides a deeper overview of the access and barriers of internet access and other supporting IT infrastructure in 10,378 public health centers throughout Indonesia.

## Conclusions

The internet plays a vital role in health care services, facilitating communication, information exchange, and access to medical resources. Despite the fact that 78% of health care centers in Indonesia have sufficient internet connectivity, challenges persist, with some areas experiencing limited access or complete absence of internet connectivity. To ensure universal availability of the internet across primary care services in Indonesia, additional measures are imperative. This study demonstrates the need for collaborative actions among government, health care workers, and telecom providers to allocate a supportive budget for sustainable internet infrastructure, including satellite-based internet services that enable strong internet connection in remote, underserved, and digitally excluded regions. Technology infrastructure should also follow, including high-quality computer systems with adequate specifications to handle health care–related tasks efficiently, as well as ensuring uninterrupted 24-hour access to electrical power to support continuous internet connectivity and operational efficiency in health care facilities. Future studies are encouraged to explore the relationship between internet quality and health care service delivery outcomes to better understand its operational impact. Additionally, longitudinal assessments could help monitor improvements over time and guide digital infrastructure investments in public health systems. Although this study does not assess clinical performance or health care outcomes, it provides crucial baseline evidence on digital infrastructure status. These insights can help guide targeted investments and future evaluations as Indonesia advances toward nationwide digital health integration.

## Supplementary material

10.2196/65940 Multimedia Appendix 1 Scoring and weighting for internet quality data analysis.

10.2196/65940 Multimedia Appendix 2 Number of Puskesmas without internet by province.

10.2196/65940 Multimedia Appendix 3 Participation rate of Puskesmas in “internet quality assessment across public health center (Puskesmas) in Indonesia.”

10.2196/65940 Multimedia Appendix 4 Distribution based on types of internet use and internet quality based on participants’ perspectives. LAN: local area network; VSAT: very small aperture terminal.

## Abbreviations

CPU: central processing unit
DTO: Digital Transformation Office
EMR: electronic medical record
ICT: information and communication technology
LAN: local area network
LMIC: low- and middle-income countries
MoH: Ministry of Health
PHC: primary health care
Puskesmas: public health centers
QoE: Quality of Experience
QoS: Quality of Service
SISMAL: Sistem Informasi Malaria
