# Supplementary material for: Assessing Internet Quality Across Public Health Centers in Indonesia: Cross-Sectional Evaluation Study
Source: JMIR Med Inform. 2025 Sep 15;13:e65940. doi: 10.2196/65940 (PMC12435787; doi:10.2196/65940)
Supplement: Multimedia Appendix 1 [file medinform-v13-e65940-s001.docx]

**Appendix 1. Scoring and Weighting for Internet Quality Data Analysis**

| **Puskesmas Internet Quality Score Weighting** | | | | | | | |
| --- | --- | --- | --- | --- | --- | --- | --- |
| **No.** | **Question** | **Weighting** | **Category** | | | **Scoring** | **Scoring x Weighting** |
| 1 | Morning Download Bandwidth Speedtest Result | 10% | Above 50 mbps | | | 4 | [Score]*0.10 |
|  |  |  | 25 - 50 mbps | | | 3 |  |
|  |  |  | 10 - 24,9 mbps | | | 2 |  |
|  |  |  | Under 10 mbps | | | 1 |  |
| 2 | Morning Upload Bandwidth Speedtest Result | 17% | Above 15 mbps | | | 4 | [Score]*0.17 |
|  |  |  | 5.1 - 14.9 mbps | | | 3 |  |
|  |  |  | 1 - 5 mbps | | | 2 |  |
|  |  |  | Under 1 mbps | | | 1 |  |
| 3 | Afternoon Download Bandwidth Speedtest Result | 9% | Above 50 mbps | | | 4 | [Score]*0.09 |
|  |  |  | 25 - 50 mbps | | | 3 |  |
|  |  |  | 10 - 24,9 mbps | | | 2 |  |
|  |  |  | Under 10 mbps | | | 1 |  |
| 4 | Afternoon Upload Bandwidth Speedtest Result | 13% | Above 15 mbps | | | 4 | [Score]*0.13 |
|  |  |  | 5.1 - 14.9 mbps | | | 3 |  |
|  |  |  | 1 - 5 mbps | | | 2 |  |
|  |  |  | Under 1 mbps | | | 1 |  |
| 5 | Type of Internet Connection (LAN, WiFi, or other) | 8% | LAN | | | 4 | [Score]*0.08 |
|  |  |  | Wifi / Wifi Access Point | | | 3 |  |
|  |  |  | Tethering/Hotspot from Handphone | | | 1 |  |
|  |  |  | No internet | | | 0 |  |
| 6 | Internet Connection Quality (LAN/WiFi) at the community health centre (Puskesmas) | 8% | No significant internet problems occurs | | | 4 | [Score]*0.08 |
|  |  |  | Occasionally experiencing internet disconnections | | | 3 |  |
|  |  |  | Sometimes experiencing internet disconnections | | | 2 |  |
|  |  |  | Always & Often experiencing internet disconnections | | | 1 |  |
|  |  |  | No Internet | | | 0 |  |
| 7 | CPU Specifications of the Laptop/Computer in use | 8% | core i7 or above | Ryzen 6 dan 7 | M1, M2, Corei7, Xeon or above | 4 | [Score]*0.08 |
|  |  |  | core i5 | Ryzen 5, Phenom II X6, Opteron 6100 series, Opteron 6200 series, Opteron 6300 series | core i5 | 3 |  |
|  |  |  | core i3 | Ryzen 3, Phenom II X2, X4, X6, Opteron 3200 series, Opteron 3300 series, Opteron 4100 series, Opteron 4200 series, Opteron 4300 series | core i3 | 2 |  |
|  |  |  | core2duo / celeron | AMD Athlon, Athlon II, Sempron, Turion, Phenom | core 2 duo | 1 |  |
| 8 | RAM Specifications of the Laptop/Computer in use | 13% | 32 GB | | | 4 | [Score]*0.13 |
|  |  |  | 16 GB | | | 3 |  |
|  |  |  | 4 - 8 GB | | | 2 |  |
|  |  |  | ≤ 2 GB | | | 1 |  |
| 9 | Availability of 24-hour Electricity at the community health centre | 8% | Yes, electricity is available 24 hours | | | 4 | [Score]*0.08 |
|  |  |  | Not 24 Hours, Sometimes there are sudden power outages under certain conditions at the Health Center | | | 3 |  |
|  |  |  | Not 24 hours, There are regular power outages, Outages NOT during the health center's operational hours | | | 2 |  |
|  |  |  | Not 24 hours, There are regular power outages, Outages YES during the health center's operational hours | | | 1 |  |
|  |  |  | No electricity | | | 0 |  |
| 10 | Availability of Backup / Alternative Power Supply | 3% | There is backup power | | | 4 | [Score]*0.03 |
|  |  |  | There is no backup power | | | 2 |  |
| 11 | Antivirus Installation Status on Laptop/Computer | 3% | Computer/Laptop installed with antivirus, latest update (November 1, 2022) | | | 4 | [Score]*0.03 |
|  |  |  | Computer/Laptop installed with antivirus | | | 3 |  |
|  |  |  | Computer/Laptop not installed with antivirus | | | 1 |  |
|  | *Sum* | *100%* |  | | |  |  |

| Level 1 | Score of 297-400 | Internet Access available with adequate/good quality |
| --- | --- | --- |
| Level 2 | Score of 188-296 | Internet Access available with sufficient quality |
| Level 3 | Score of 0-187 | Internet Access available but not sufficient |
| Level 4 | Score of 0 | No Internet Access |
