# Supplementary material for: Assessing Internet Quality Across Public Health Centers in Indonesia: Cross-Sectional Evaluation Study
Source: JMIR Med Inform. 2025 Sep 15;13:e65940. doi: 10.2196/65940 (PMC12435787; doi:10.2196/65940)
Supplement: Multimedia Appendix 2 [file medinform-v13-e65940-s002.docx]

**Appendix 2. Number of Puskesmas Without Internet by Province**


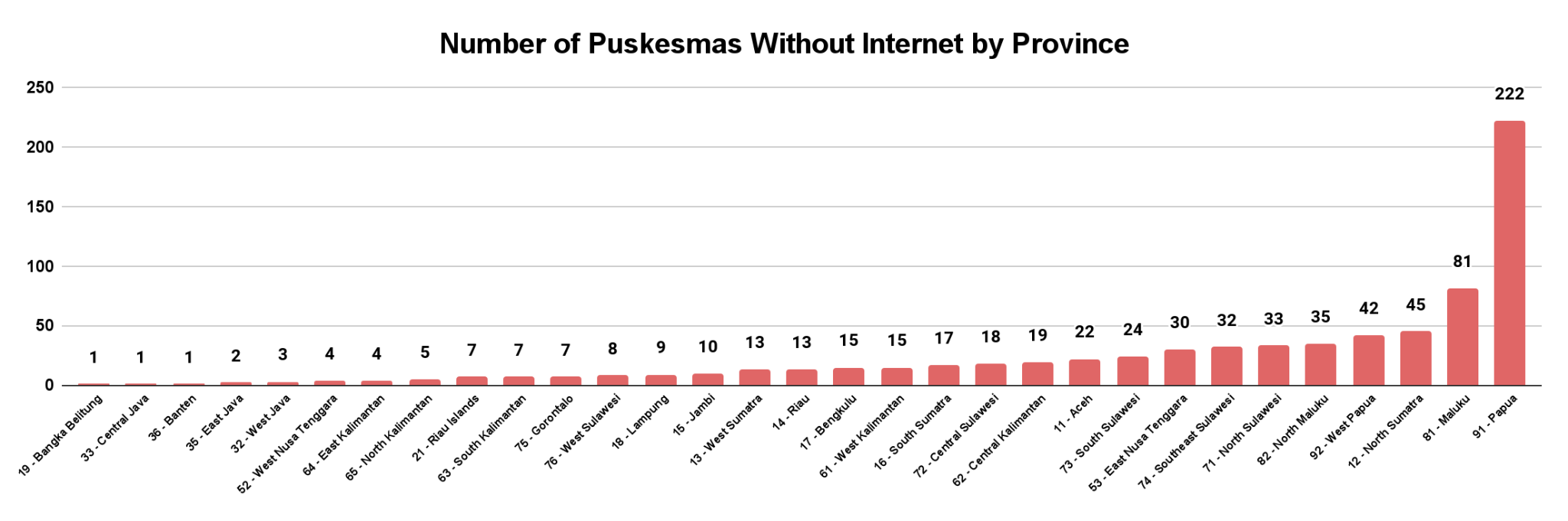


| **No** | **Province** | **Puskesmas Without Internet** | **Total of Puskesmas** | **% Puskesmas Without Internet** |
| --- | --- | --- | --- | --- |
| 1 | Aceh | 22 | 362 | 6.08% |
| 2 | North Sumatra | 45 | 617 | 7.29% |
| 3 | West Sumatra | 13 | 280 | 4.64% |
| 4 | Riau | 13 | 238 | 5.46% |
| 5 | Jambi | 10 | 207 | 4.83% |
| 6 | South Sumatra | 17 | 348 | 4.89% |
| 7 | Bengkulu | 15 | 179 | 8.38% |
| 8 | Lampung | 9 | 318 | 2.83% |
| 9 | Bangka Belitung Islands | 1 | 64 | 1.56% |
| 10 | Riau Islands | 7 | 93 | 7.53% |
| 11 | DKI Jakarta | 0 | 315 | 0.00% |
| 12 | West Java | 3 | 1,100 | 0.27% |
| 13 | Central Java | 1 | 880 | 0.11% |
| 14 | DI Yogyakarta | 0 | 121 | 0.00% |
| 15 | East Java | 2 | 972 | 0.21% |
| 16 | Banten | 1 | 247 | 0.40% |
| 17 | Bali | 0 | 120 | 0.00% |
| 18 | West Nusa Tenggara | 4 | 176 | 2.27% |
| 19 | East Nusa Tenggara | 30 | 432 | 6.94% |
| 20 | West Kalimantan | 15 | 248 | 6.05% |
| 21 | Central Kalimantan | 19 | 204 | 9.31% |
| 22 | South Kalimantan | 7 | 241 | 2.90% |
| 23 | East Kalimantan | 4 | 188 | 2.13% |
| 24 | North Kalimantan | 5 | 57 | 8.77% |
| 25 | North Sulawesi | 33 | 199 | 16.58% |
| 26 | Central Sulawesi | 18 | 218 | 8.26% |
| 27 | South Sulawesi | 24 | 472 | 5.08% |
| 28 | Southeast Sulawesi | 32 | 302 | 10.60% |
| 29 | Gorontalo | 7 | 93 | 7.53% |
| 30 | West Sulawesi | 8 | 98 | 8.16% |
| 31 | Maluku | 81 | 228 | 35.53% |
| 32 | North Maluku | 35 | 148 | 23.65% |
| 33 | Papua | 222 | 454 | 48.90% |
| 34 | West Papua | 42 | 163 | 25.77% |
|  | **Total** | **745** | **10,382** | **7.18%** |
