# Supplementary material for: Assessing Internet Quality Across Public Health Centers in Indonesia: Cross-Sectional Evaluation Study
Source: JMIR Med Inform. 2025 Sep 15;13:e65940. doi: 10.2196/65940 (PMC12435787; doi:10.2196/65940)
Supplement: Multimedia Appendix 3 [file medinform-v13-e65940-s003.docx]

**Appendix 3. Participation Rate of Puskesmas in “Internet Quality Assessment across Public Health Centre (Puskesmas) in Indonesia”**

| **No** | **Province** | **Total of Puskesmas** | **Participating Puskesmas** | **% Participation Rate** |
| --- | --- | --- | --- | --- |
| 1 | Aceh | 362 | 361 | 99.72% |
| 2 | North Sumatra | 617 | 617 | 100.00% |
| 3 | West Sumatra | 280 | 279 | 99.64% |
| 4 | Riau | 238 | 238 | 100.00% |
| 5 | Jambi | 207 | 207 | 100.00% |
| 6 | South Sumatra | 348 | 348 | 100.00% |
| 7 | Bengkulu | 179 | 179 | 100.00% |
| 8 | Lampung | 318 | 318 | 100.00% |
| 9 | Bangka Belitung Island | 64 | 64 | 100.00% |
| 10 | Riau Islands | 93 | 93 | 100.00% |
| 11 | DKI Jakarta | 315 | 315 | 100.00% |
| 12 | West Java | 1,100 | 1,100 | 100.00% |
| 13 | Central Java | 880 | 880 | 100.00% |
| 14 | DI Yogyakarta | 121 | 121 | 100.00% |
| 15 | East Java | 972 | 972 | 100.00% |
| 16 | Banten | 247 | 247 | 100.00% |
| 17 | Bali | 120 | 120 | 100.00% |
| 18 | West Nusa Tenggara | 176 | 176 | 100.00% |
| 19 | East Nusa Tenggara | 432 | 432 | 100.00% |
| 20 | West Kalimantan | 248 | 248 | 100.00% |
| 21 | Central Kalimantan | 204 | 204 | 100.00% |
| 22 | South Kalimantan | 241 | 241 | 100.00% |
| 23 | East Kalimantan | 188 | 188 | 100.00% |
| 24 | North Kalimantan | 57 | 57 | 100.00% |
| 25 | North Sulawesi | 199 | 199 | 100.00% |
| 26 | Central Sulawesi | 218 | 217 | 99.54% |
| 27 | South Sulawesi | 472 | 472 | 100.00% |
| 28 | Southeast Sulawesi | 302 | 302 | 100.00% |
| 29 | Gorontalo | 93 | 93 | 100.00% |
| 30 | West Sulawesi | 98 | 98 | 100.00% |
| 31 | Maluku | 228 | 227 | 99.56% |
| 32 | North Maluku | 148 | 148 | 100.00% |
| 33 | Papua | 454 | 454 | 100.00% |
| 34 | West Papua | 163 | 163 | 100.00% |
|  | **Total** | **10,382** | **10,378** | **99.96%** |
