# Supplementary material for: Assessing Internet Quality Across Public Health Centers in Indonesia: Cross-Sectional Evaluation Study
Source: JMIR Med Inform. 2025 Sep 15;13:e65940. doi: 10.2196/65940 (PMC12435787; doi:10.2196/65940)

Distribution based on types of internet use and internet quality based on participants’ perspectives. LAN: local area network; VSAT: very small aperture terminal.


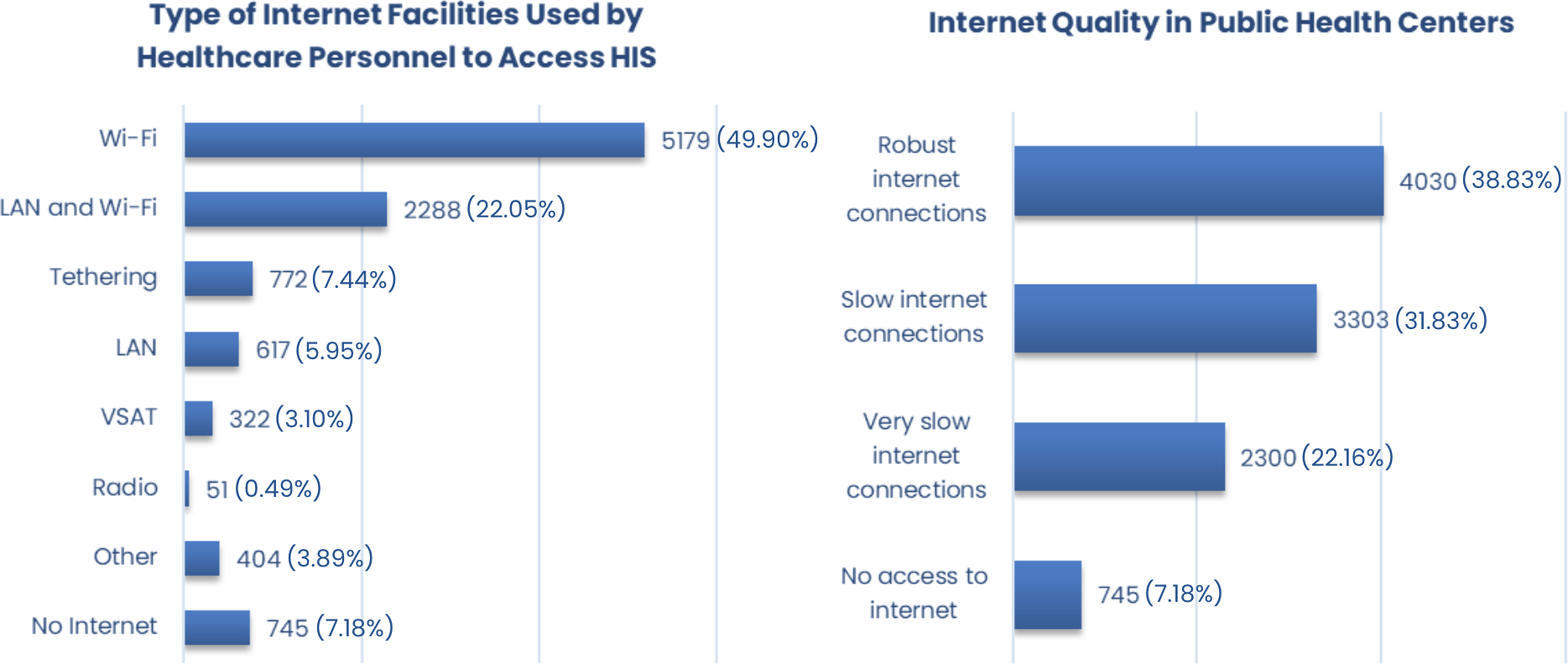

Supplement: Multimedia Appendix 4 [file medinform-v13-e65940-s004.docx]
